# Typical Asthmatic Presentation of Congenital Vascular Ring Can Masquerade a General Physician

**DOI:** 10.1155/2013/934790

**Published:** 2013-02-07

**Authors:** Naveen Swami, Georgey Koshy, Maan Jamal, Thair S. Abdulla, Abdulaziz Alkhulaifi

**Affiliations:** ^1^Department of Cardiothoracic Surgery, Heart Care Centre, Al Ahli Hospital, 2nd Floor, Bin Omran, P.O. Box 6401, Doha, Qatar; ^2^Department of Cardiology, Heart Care Centre, Al Ahli Hospital, Doha, Qatar; ^3^Department of Radiology, Al Ahli Hospital, Doha, Qatar; ^4^Department of Pulmonary Medicine & Intensive Care Unit, Al Ahli Hospital, Doha, Qatar; ^5^Department of Cardiothoracic Surgery, Heart Hospital, Hamad Medical Corporation, Doha, Qatar

## Abstract

A 24-year-old woman was referred to pulmonologist with worsening breathlessness and wheeze. During childhood, she was diagnosed with asthma and subsequent exacerbations were treated with bronchodilators for many years. The chest X-ray and a spirometry testing raised a doubt of extrinsic tracheal compression and a subsequent enhanced chest CT (computerized tomogram) scan confirmed a right-sided aortic arch and a vascular ring anomaly compressing the trachea. Standard surgical division of ligamentum arteriosum was able to relieve the trachea and so the symptoms.

## 1. Introduction

Vascular rings are congenital malformations that result from abnormal development of the aortic arch complex and can cause encirclement of the trachea and oesophagus [1]. Symptoms tend to occur early in life, mainly as a result in airway compression [2]. There have been few cases in the literature of adult vascular rings presenting with asthma-like symptoms [3] and indeed respiratory arrest on one occasion [4].

We report on an interested case of a young woman who had been treated several years as an asthmatic on clinical background, but, in fact the underlying pathology has been a tracheal compression caused by congenital vascular ring.

## 2. Case

A 24-year-old woman came first time to the pulmonary clinic complaining of progressive breathlessness on exertion, chest tightness, audible wheezing, and dry cough for the past few months. Since her late childhood, she was diagnosed as being asthmatic with intermittent exacerbations. She had received inhalational bronchodilators, antibiotics, and several courses of corticosteroids over the past few years but, never subjected to any spirometry testing. The worsening symptoms, in spite of aggressive medical treatment, compelled her to consult a pulmonologist.

**Figure 1:**
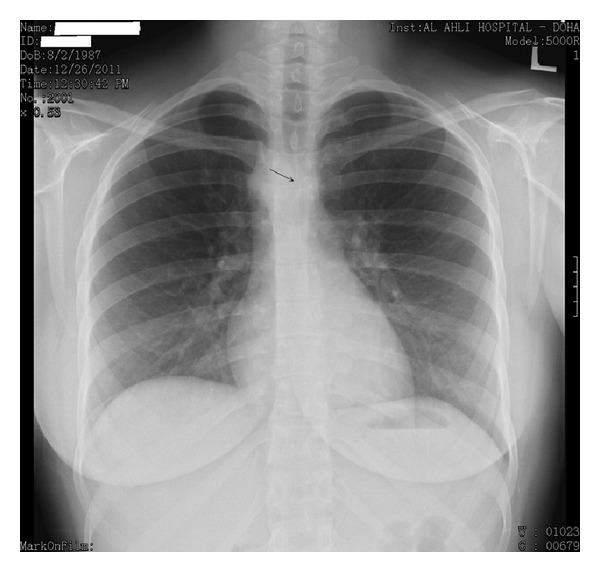
A posteroanterior view of chest X-ray showing an abnormal distal tracheal air shadow (arrow) and the absent left aortic knuckle.

**Figure 2:**
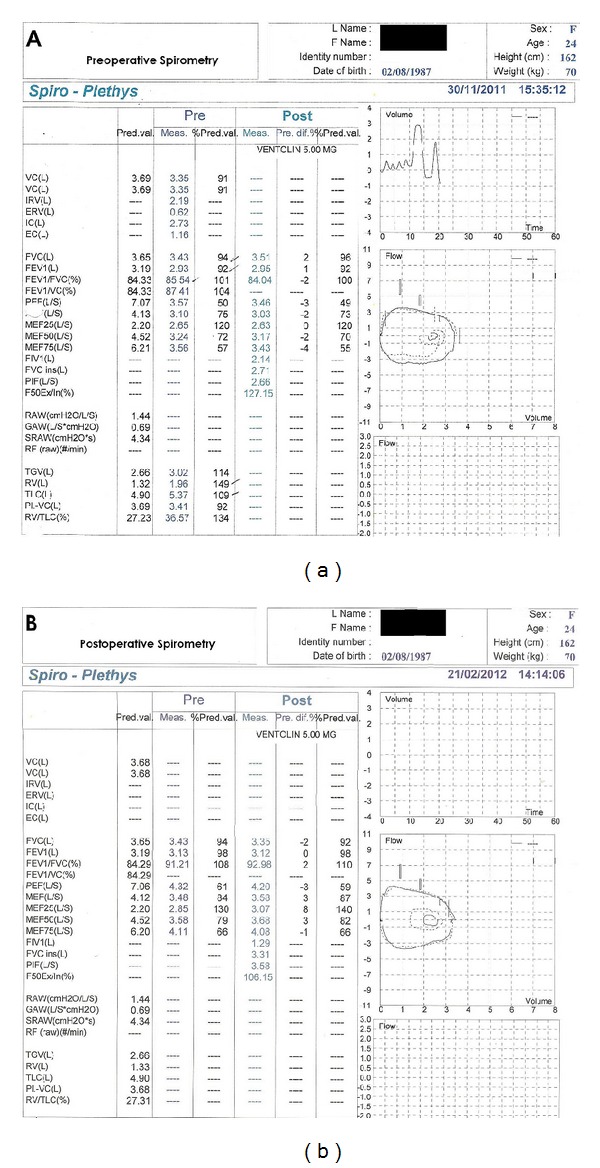
(a) Preoperative flow-volume loop demonstrating flattening of the inspiratory and expiratory segments (b) Flow-volume loop, after 8 weeks of surgery, showing slightly improved inspiratory and expiratory phases.

Her physical examination revealed mild inspiratory and expiratory wheezes over the upper anterior chest with stridor, though her pulse oximetry showed 100% oxygen saturation on room air. Otherwise, the rest of the physical examination was normal. A routine chest roentgenogram showed absent aortic knuckle on the left side with a right-sided aortic arch and an abnormal distal tracheal air shadow (Figure 1). In addition to X-ray findings, the flattening of both inspiratory and expiratory segment of flow-volume loop during spirometry raised a possibility of aortic arch and branch vessel anomaly (Figure 2(a)).

**Figure 3:**
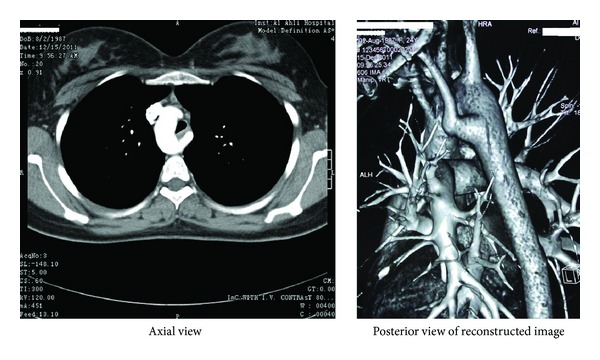
A collated picture of chest CT scan with an image of axial view and a reconstructed image with posterior facing showing a right-sided aortic arch, an aberrant origin of left Subclavian artery (Kommerell's diverticulum) behind the oesophagus which was obscured due to compression.

Subsequent, spiral CT of chest with 3-dimensional reconstruction (Figure 3) showed a right-sided aortic arch with 3 branch arteries. The left carotid and right carotid arteries were originating as the first and second branches, respectively, whereas the left subclavian, the 3rd branch with aberrant origin (Kommerell's diverticulum), was originating from the distal portion of the arch and traversing the space behind the esophagus. The descending aorta was only slightly to the right of the midline. A deviation to the left and compression of the trachea and esophagus were seen as a result of the right aortic arch and the anomalous origin and course of left subclavian artery. The ductus arteriosus could not be visualized, possibly due to fibrous obliteration.

She underwent a standard left posterolateral thoracotomy, and through the bed of the 4th rib, chest was entered. The ligamentum arteriosum, connecting the origin of the left subclavian artery and the left pulmonary artery, constituted the majority of compression by culprit vascular ring. Its division, over sewn and with subsequent mobilization of the surrounding structures, successfully relieved the constriction.

- The patient made a remarkable improvement after surgery and continued to improve with gradual disappearance of her exertional breathlessness, wheeze, and stridor.
- At 8 weeks, she underwent a repeat Respiratory Function Test which has revealed slight improvement as of increase in FVC and FEV1/FVC% and reshaping of flow-volume loop (Figure 2(b)).

## 3. Discussion

Vascular rings compressing the trachea and the esophagus are congenital malformations that result from abnormal development of the embryonic aortic arch complex. The usual presentation is that of respiratory distress in the neonatal period. However, there have been few cases diagnosed in adulthood with varying modes of presentations, such as asthma [3], dyspnoea, and dysphagia [1, 2]. The commonest two forms of the vascular rings are associated with right aortic arch with either aberrant left subclavian artery or aberrant innominate artery with ligamentum arteriosum completing the vascular ring. Hence, the treatment, when indicated is based on division of the ligament.

In this case, asthma had been diagnosed since childhood and she received several courses of steroids treatment and bronchodilators. One suspect that she had a number of chest X-ray performed, but it is possible that due to the lack of prominence of the right aortic arch and the low index of suspicion, this anomaly could not be suspected. Unfortunately, she did not undergo any pulmonary function testing prior to pulmonologist consultation which could have given a strong suspicion. 

It is always imperative to have anatomical details of aortic anomalies as these could determine the surgical approach for correction. As mentioned earlier, in this woman, the anomalous origin and course of left subclavian artery and the ligamentum arteriosum were the main reason for the extrinsic compression of trachea and esophagus. A left thoracotomy was the approach for correction of this anomaly with immediate improvement in symptoms after surgery.

## Abbreviations

CT: Computerized tomogram
FEV1: Forced expiratory volume in first second
FVC: Forced vital capacity.
